# The Use of Two Culturing Methods in Parallel Reveals a High Prevalence and Diversity of* Arcobacter* spp. in a Wastewater Treatment Plant

**DOI:** 10.1155/2016/8132058

**Published:** 2016-11-17

**Authors:** Arturo Levican, Luis Collado, Maria José Figueras

**Affiliations:** ^1^Escuela de Tecnología Médica, Facultad de Ciencias, Pontificia Universidad Católica de Valparaíso, Avenida Universidad 330, 2373223 Valparaíso, Chile; ^2^Instituto de Bioquímica y Microbiología, Facultad de Ciencias, Universidad Austral de Chile, Valdivia, Chile; ^3^Unitat de Microbiologia, Departament de Ciències Mediques Bàsiques, Facultat de Medicina i Ciències de la Salut, IISPV, Universitat Rovira i Virgili, Reus, Spain

## Abstract

The genus* Arcobacter* includes species considered emerging food and waterborne pathogens. Despite* Arcobacter* has been linked to the presence of faecal pollution, few studies have investigated its prevalence in wastewater, and the only isolated species were* Arcobacter butzleri* and* Arcobacter cryaerophilus*. This study aimed to establish the prevalence of* Arcobacter* spp. at a WWTP using in parallel two culturing methods (direct plating and culturing after enrichment) and a direct detection by m-PCR. In addition, the genetic diversity of the isolates was established using the ERIC-PCR genotyping method. Most of the wastewater samples (96.7%) were positive for* Arcobacter* and a high genetic diversity was observed among the 651 investigated isolates that belonged to 424 different ERIC genotypes. However, only few strains persisted at different dates or sampling points. The use of direct plating in parallel with culturing after enrichment allowed recovering the species A.* butzleri*, A.* cryaerophilus*,* Arcobacter thereius*,* Arcobacter defluvii*,* Arcobacter skirrowii*,* Arcobacter ellisii*,* Arcobacter cloacae*, and* Arcobacter nitrofigilis*, most of them isolated for the first time from wastewater. The predominant species was A.* butzleri*, however, by direct plating predominated A.* cryaerophilus*. Therefore, the overall predominance of A.* butzleri* was a bias associated with the use of enrichment.

## 1. Introduction

The genus* Arcobacter* is included together with* Campylobacter* and* Helicobacter* in the family* Campylobacteraceae*, and all of these genera include species that might be pathogenic to humans and animals [1, 2].* Arcobacter butzleri* is the fourth most common* Campylobacter*-like organism isolated from the stool of human patients with diarrhoea in two independent studies carried out in France [3] and Belgium [4]. Furthermore, in a recent study,* Arcobacter* species was the fourth most common pathogen group isolated from faecal samples from persons with acute enteric disease [5]. It has been demonstrated that the presence of* Arcobacter* in water correlates with the presence of faecal pollution [2]. In this sense,* Arcobacter* has been recovered in three outbreaks in which drinking water was contaminated with sewage ([2] and references therein). Food products, especially meat, shellfish, and milk, have also been found contaminated with bacteria of this genus, mainly with* A. butzleri* [2, 6]. Considering this, the* International Commission on Microbiological Specifications for Foods* has defined* A. butzleri* as a serious hazard to human health [6], and it has been identified as an important zoonotic agent to humans and animals ([2] and references therein).

Disposal of sewage is a critical issue in modern cities that normally deliver it to receiving waters after treatment at wastewater treatment plants (WWTPs). This treatment is aimed at reducing degradable organic matter under controlled conditions before it is discharged into natural bodies of water [7]. However, conventional primary and secondary treatments per se (without disinfection steps) do not eliminate the pathogens present in the water and as a result WWTP outflows contain a lot of microbes that are potentially pathogenic to humans and animals.

The presence of* Arcobacter* in water, including sewage from WWTPs, has been reported in several studies [2, 8–13]. In those studies,* Arcobacter* spp. were isolated in 40% to 100% of the samples studied, using different culture media and protocols, and were found in 66% to 100% of the samples when direct detection by molecular techniques was used [2, 10–14]. Three studies have investigated the presence of* Arcobacter* in WWTPs after using different treatments [8–10]. Despite different results being obtained all the studies showed the presence of these bacteria at all points of the treatment, including the water outflow. Furthermore, using pyrosequencing of the V6 hypervariable region of 16S rRNA gene,* Arcobacter* were found to be one of the predominant taxa in WWTPs in Milwaukee (USA) in contrast to their scarcity in surface waters [15]. In fact, considering those results,* Arcobacter* spp. were selected as “sewer signature microbes” together with* Acinetobacter* and* Trichococcus* (the most common taxa in sewage) in the detection of sewage contamination of surface waters [16].

Studies on wastewater samples using conventional culture protocols that included an enrichment step in a selective broth found that* A. butzleri* was more predominant than* A. cryaerophilus* [2, 8–12]. However, it has been suggested that growth of some* Arcobacter* species may be enhanced in the enrichment broth, which can mask other species, leading to a bias in the estimation of the diversity [17]. On the other hand, the best atmosphere of incubation (aerobic or microaerophilic) for arcobacters has not yet been determined, and half of the studies have used aerobic conditions [2]. Furthermore, only one study so far has compared the effect of both atmosphere incubation conditions on the recovery of* Arcobacter*, but it did not reach clear conclusions [11].

The genetic diversity of* Arcobacter* in sewage has seldom been studied and methods used include random amplification of polymorphic DNA [12] and enterobacterial repetitive intergenic consensus (ERIC-PCR) [18]. Results showed a wide range of genotypes, as happens in samples from other environments [2].

The objective of this survey is to establish the prevalence and genetic diversity of* Arcobacter* spp. in a WWTP using two culturing approaches (direct plating and culturing after enrichment) and comparing the recovery under aerobic or microaerophilic conditions, using direct detection by m-PCR in parallel.

## 2. Materials and Methods

### 2.1. Samples and Water Processing

**Figure 1:**
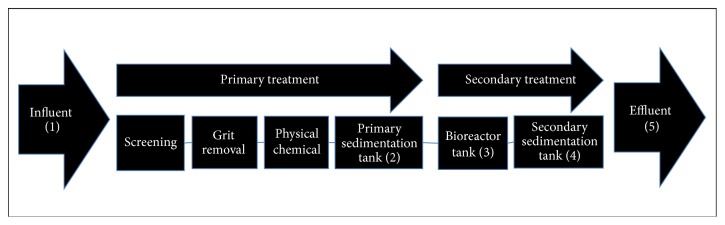
Simplified scheme of the wastewater treatment plant indicating the five sampling points (1 to 5).

The wastewater samples were collected on six sampling dates (April, June, and October 2009 and May, June, and September 2011) from five sampling points (Figure 1) at the WWTP in Reus, Spain, that produced nondisinfected secondary treated wastewater. The sampling points were located in the influent and effluent water in the primary and secondary sedimentation tanks and in the bioreactor tank. Samples were collected into 2-litre sterile polypropylene bottles, which were then chilled in ice during transport. Microbiological assays began on the same day as sampling.

From each water sample 200 mL was filtered through a 0.45 *μ*m membrane filter (47 mm diameter) in duplicate (Millipore Corp., Bedford, MA, USA). Then the filters were rolled and one of them was introduced into a tube containing 1 mL distilled water and vigorously mixed by vortexing. The other filter was then introduced into a tube containing 9 mL of* Arcobacter*-CAT broth (*Arcobacter*-enrichment broth supplemented with the CAT antibiotic supplement, i.e., Cefoperazone, amphotericin B and teicoplanin, and oxoid, Basingstoke, UK) and incubated aerobically (30°C, 48 to 72 h).

### 2.2. Molecular Detection

For molecular detection, 400 *μ*L of water from the 2 tubes was centrifuged at 12,000 rpm min^−1^ and the obtained pellet was resuspended and washed 3 times with Milli-Q sterile water. Afterwards, DNA was extracted by using the InstaGene™ DNA Purification Matrix (Bio-Rad Laboratories, Hercules, CA), and direct detection of* Arcobacter* was carried out using the primers and conditions of the m-PCR designed by Houf et al. [19] for the detection of* A. butzleri*,* A. cryaerophilus,* and* A. skirrowii*. The procedure included initial denaturation for 2 min at 94°C followed by 32 cycles of denaturation for 45 sec at 94°C, annealing for 45 sec at 61°C, and chain extension for 60 sec at 72°C and a final extension for 5 min at 72°C. The PCRs were carried out in a reaction mixture containing 1 *μ*L of DNA extract, 1.5 U of* Taq* DNA polymerase (Invitrogen, Carlsbad, CA), 0.2 mM of each deoxyribonucleotide triphosphate, 50 pmol of each primer set (Invitrogen, Carlsbad, CA), and 1.3 mM of MgCl_2_.

For molecular detection, 400 *μ*L of water from the 2 tubes was centrifuged at 12,000 rpm min^−1^ and the obtained pellet was resuspended and washed 3 times with Milli-Q sterile water. Afterwards, DNA was extracted by using the InstaGene DNA Purification Matrix (Bio-Rad Laboratories, Hercules, CA), and direct detection of* Arcobacter* was carried out using the primers and conditions of the m-PCR designed by Houf et al. [19] for the detection of* A. butzleri, A. cryaerophilus,* and* A. skirrowii*. The procedure included initial denaturation for 2 min at 94°C followed by 32 cycles of denaturation for 45 sec at 94°C, annealing for 45 sec at 61°C, and chain extension for 60 sec at 72°C and a final extension for 5 min at 72°C. The PCRs were carried out in a reaction mixture containing 1 *μ*L of DNA extract, 1.5 U of* Taq* DNA polymerase (Invitrogen, Carlsbad, CA), 0.2 mM of each deoxyribonucleotide triphosphate, 50 pmol of each primer set (Invitrogen, Carlsbad, CA), and 1.3 mM of MgCl_2_.

### 2.3. Culturing Procedure

For the direct detection by culturing, 200 *μ*L of water from the nonenriched tube was transferred onto the surface of a 0.45 *μ*m membrane filter (47 mm diameter), placed on blood agar medium (Trypticase Soy Agar, oxoid, Basingstoke, UK, supplemented with 5% sheep blood), and allowed to filter passively under ambient conditions for 30 min [5]. For culturing after enrichment, 200 *μ*L of incubated* Arcobacter*-CAT broth (enrichment broth) was transferred onto the surface of a 0.45 *μ*m membrane filter (47 mm diameter), placed on blood agar medium, and allowed to filter as described above. The filters were then removed and the plates were incubated (30°C, 48 to 72 h) under aerobic conditions.

For samples collected in 2011, direct and postenrichment culturing was also processed in duplicate in order to allow the parallel incubation under aerobic and microaerophilic conditions. The microaerophilic conditions (oxygen, 6% to 16%; carbon dioxide, 2% to 10%; and nitrogen, 80%) were generated by using GasPak EZ campy container system sachets (Becton Dickinson, Sparks, MD, USA).

### 2.4. *Arcobacter* Isolation, Genotyping, and Identification

From each positive sample, eight small, colourless or beige to off-white, translucent colonies were picked, streaked to purity, and confirmed as presumptive arcobacters on the basis of their response to phenotypic tests (i.e., gram negative slightly curved rods that were positive for oxidase and motility). All isolates were genotyped using the ERIC-PCR technique, using the Houf et al. [17] protocol for* Arcobacter*. DNA was extracted as described above and the concentration of each DNA template was determined using the GeneQuant pro (Amersham Biosciences, Cambridge, England) at A260 and adjusted to 25 ng mL^−1^. Gel images were saved as TIFF files, normalized with the 100 bp DNA Ladder (Invitrogen), and further analysed by Bionumerics software, version 6.5 (Applied Maths, Belgium). Patterns with one or more different bands were considered different genotypes [17].

All strains (1 representative of each genotype) were finally identified using in parallel two molecular identification methods, the m-PCR described above for the direct detection [19] and the 16S rDNA-RFLP [1, 20]. When identification was not possible with these methods or discordant results were obtained, the* rpoB* and/or 16S rRNA genes were sequenced using primers and conditions previously described [1].

### 2.5. Counting of* Arcobacter*

Direct counting of* Arcobacter* was carried out from all wastewater samples collected in 2011 as previously described [21, 22]. In brief, water samples were tenfold diluted in 0.1% peptone water (oxoid, Basingstoke, UK), from 10^0^ to 10^−8^ and then 100 *μ*L of each tenfold dilution was inoculated onto* Arcobacter *selective isolation agar plate (24 g litre^−1^ *Arcobacter *broth, oxoid, Basingstoke, UK; 12 g litre^−1^, Agar Technical No. 3, oxoid, Basingstoke, UK; supplemented with 100 mg litre^−1^ 5-fluorouracil, 100 mg litre^−1^ cycloheximide, 10 mg litre^−1^, amphotericin B, 16 mg litre^−1^ cefoperazone, 32 mg litre^−1^ novobiocin, and 64 mg litre^−1^ trimethoprim, Sigma, USA) [23]. All plates were then incubated for 48 h at 30°C under microaerophilic conditions. After incubation, plates were checked for typical bluish colonies using Henry transillumination and the colony forming units (CFU) were counted and then informed as CFU mL^−1^ [22, 23]. The tenfold dilutions prepared in peptone water were used to enumerate* Arcobacter* using the MPN method. Briefly, each of the 5 dilutions (1, 0.1, 0.001, 0.0001, and 0.00001 mL of the original sample) was inoculated in 5 tubes containing* Arcobacter*-CAT broth for the MPN calculation as previously described by Levican [1]. The broths were incubated for 48 h under aerobic conditions and then 100 *μ*L of each tube was inoculated by passive filtration onto 5% sheep blood agar plates and incubated under the same conditions. The MPN of* Arcobacter *in 100 mL was estimated from the obtained combination of positive tubes using the MPN CALCULATOR Software (Curiale M, 2004 available from http://www.i2workout.com/mcuriale/mpn/).

In order to confirm that typical colonies obtained by direct counting or from the MPN positive tubes belonged to* Arcobacter* spp., a representative number per plate was randomly selected to be identified by the molecular methods described above, that is, m-PCR [19] and 16S rDNA-RFLP [1, 20].

### 2.6. Statistical Analyses

The proportions obtained using different methods were compared using the *Z* test and a *P* value < 0.05 was considered as statistically significant.

## 3. Results and Discussion

### 3.1. Prevalence and Diversity of* Arcobacter* Species

*Arcobacter* spp. were recovered from 29 of the 30 samples (96.7%), from which 651 isolates recovered by culturing were confirmed to belong to the genus* Arcobacter* (Table 1). Those isolates were genotyped with ERIC-PCR and their patterns indicated that they belonged to 424 different genotypes, so the global genetic diversity was 65.1% (Table 1). In previous studies that used different culture media and protocols, the prevalence of* Arcobacter* spp. from wastewater samples ranged from 40% to 100% [10–14, 18]. When a genotyping method was applied, a high genetic diversity was observed. For example, Collado et al. [18] report that 90.2% of the isolates belonged to different ERIC-PCR genotypes, while González et al. [12] found that all of their isolates belonged to different RAPD-PCR genotypes. This high genetic diversity has previously been explained by possible multiple sources of contamination and/or as a consequence of genomic rearrangement [12, 18].

**Figure 2:**
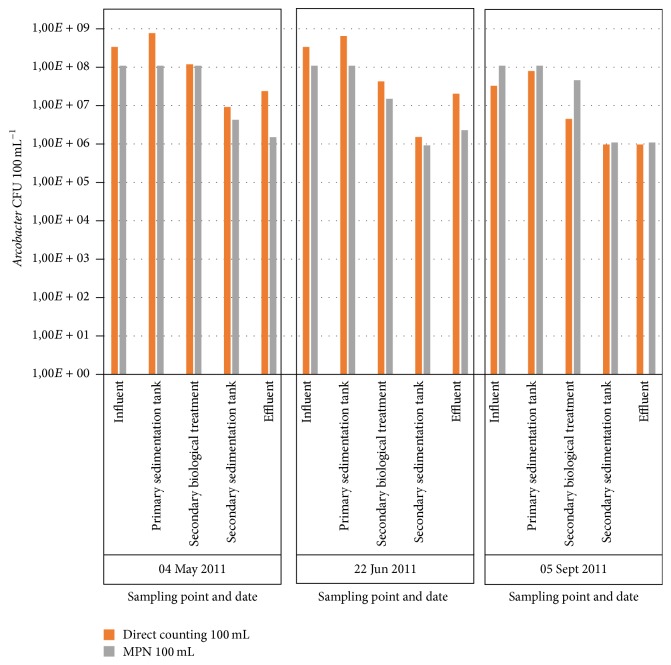
Amount of* Arcobacter* found in the different sampling points by date of sampling.

*Arcobacter* spp. were isolated from all sampling points, with the exception of only one sample taken at the water outflow (Table 2). The amount of arcobacters showed a decrease of at least 2 logarithms from the influent to the effluent of the WWTP and both enumeration methods showed similar results (Figure 2). The densities of* Arcobacter* found in the effluent water in our study are similar to the results shown in another study performed from the same WWTP [21, 24]. No seasonal variation was observed among results (Figure 2).

On the other hand, when the 424 genotypes were analysed with the Bionumerics software, only 4 of them (0.9%) were coincidentally recovered from different sampling points at the same time or on different samplings days (data not shown). This indicates that most of the* Arcobacter* strains do not persist over the time in the WWTP. As in previous studies [8, 10],* Arcobacter* was present at all sampling points suggesting that conventional wastewater treatment is not able to completely remove these bacteria.

A total of 8* Arcobacter* spp. were recovered in this study among the 424 strains, the most prevalent being* A. butzleri* and* A. cryaerophilus*, which together accounted for 94.8% (*n* = 402) of strains. Both species showed a similar genetic diversity (69.8% and 66.4%, resp.; Table 1). In a previous study in river water impacted by sewage effluents [16],* A. cryaerophilus* had a slightly wider diversity (95.2%) than* A. butzleri* (90.2%). The remaining 22 strains (5.2%) belonged to 6 species (Table 1); two of them were new species recovered for the first time from these samples and were described elsewhere, that is,* A. defluvii* [10] and* A. cloacae* [1, 21]. To our knowledge this is the first time that the other 3 species,* A. nitrofigilis*,* A. thereius*, and* A. ellisii*, have been isolated from sewage. The species* A. thereius *had been isolated previously from animal faeces and porcine abortions [22, 25] and has very recently been reported from the faeces of patients with diarrhoea in Belgium [5]. However,* A. nitrofigilis* has so far only been genetically identified from mussels [2] since its description from the roots of a salt marsh plant [26] but* A. ellisii* has never again been isolated since its description from shellfish [27]. It is noticeable that the 8* Arcobacter* spp. recovered in the present study have also been recovered from mussels in a recent study [28]. In this sense, wastewater may be the source of contamination of seawater with these bacteria in the shellfish harvesting area from where they can be concentrated in mussels by filtering. Therefore, our results also support the previous suggestion that potential pathogenic arcobacters enter seawater with sewage-polluted fresh water [2]. The two molecular identification methods used in this study [17, 18] showed the same results for 402 of the 424 strains (94.8%), 247 of them (58.3%) being* A. butzleri*, 150 (35.4%) being* A. cryaerophilus,* and 5 (1.2%) being* A. skirrowii* (Table 1). The other 22 strains (5.2%) gave different results with the two methods and their identity was confirmed by sequencing the* rpoB* and 16S rRNA genes (Table 1). Several of the available detection and identification methods for* Arcobacter* spp. have failed to recognize all known species or have confused them with the most common ones [29]. In this regard, the recognition of such a high number of different species in our study confirms the previous suggestion that the known diversity of* Arcobacter* spp. in different environments will become more precise as reliable identification methods are applied [29].

### 3.2. Detection Using m-PCR and Culturing Methods

Of the 30 samples studied, 28 (86.7%) were positive by direct plating, 29 (93.3%) by postenrichment, and 19 (63.3%) by direct m-PCR, and as indicated above only one sample taken from the WWTP outflow was negative by all three methods (Table 2). However, 15 of the 15 samples (100%) tested by m-PCR after enrichment were positive (Table 2). Therefore, comparing those methods, direct detection by m-PCR [19] performed worse (Table 2) than the other two. Previous studies that have investigated* Arcobacter* in wastewater by the same m-PCR detection method from the enrichment broth reported the same number of positive samples as by culturing [2, 11] or a higher number of positive samples by m-PCR (100%) than by culturing (45.5%) [12]. The bad performance of direct detection by m-PCR from the samples studied could be explained by different factors that were not controlled for in the present study, that is, the presence of inhibitors and the low concentration of the* Arcobacter* spp. in relation to the sensitivity of the m-PCR method for the detection of the different species. It is clear that the enrichment step improves growth, which might increase the level of target cells and thus the percentage of detection. Despite that, it has been demonstrated by Ho et al. [30] that the detection of the different species by m-PCR is biased when applied after the enrichment step. Regarding that, the latter authors suggested that the species that grow faster in enrichment are more easily detected. However, that study did not determine whether this behaviour was due to the different concentrations of the bacteria cells in the mixtures that could be under the detection limit of the method established by Houf et al. [19] at 10^3^ cfu mL^−1^.

Regarding the species detected when using this m-PCR method [19], some of them might be underestimated because the method was specifically created to detect only* A. butzleri*,* A. cryaerophilus,* and* A. skirrowii *and we know that* A. cloacae*, for example, produces the same amplicon expected for* A. cryaerophilus* (257 bp) and* A. defluvii* a very similar one (~230 bp) [2] while* A. nitrofigilis* produces the amplicon expected for* A. skirrowii* (625 bp) [2, 29].

When comparing the performance of different incubation conditions, similar results were observed; that is, 45.7% of the strains were recovered under aerobic conditions and 45.4% under microaerophilic conditions (Table 3). In both cases, the predominant species were* A. butzleri* followed by* A. cryaerophilus*, with no significant difference between results. As commented in Introduction, although about half of the existing studies used aerobic conditions for incubation ([2] and references therein) this is a poorly explored aspect and the only study to assess this gave inconclusive results [11]. Based on the results obtained in our study, the use of microaerophilic conditions seems not to be justified, considering that aerobic conditions yielded almost the same results, and is cheaper and easier to carry out.

In relation to the comparative performance of culturing methods and independently of the incubation conditions (data not shown), direct plating obtained a higher, although not significant, number of strains (*n* = 218) than postenrichment (*n* = 189) as shown in Table 4. The predominant species isolated by each method were different (Table 4); that is, the most abundant species recovered under direct plating conditions were* A. cryaerophilus* (50%) and* A. butzleri* (46.8%). However,* A. butzleri* was the most frequently isolated under postenrichment culturing conditions (74.1%) followed by far by* A. cryaerophilus* (19%; Table 4). The species* A. thereius*,* A. skirrowii,* and* A. defluvii* were isolated by both methods, whereas* A. nitrofigilis *and* A. ellisii *were recovered only by direct plating and* A. cloacae* only by postenrichment (Tables 2 and 4). Contrary to that, previous studies in wastewater have shown lower species diversity with* A. butzleri* and/or* A. cryaerophilus* being the only species recovered [2, 8, 9, 11, 12, 18]. This lower diversity has probably been originated as a result of the lower number of isolates investigated and the applied methodology including only an enrichment step but no direct plating. In fact, if only culturing after enrichment was carried out in our study,* A. butzleri *would be 3.9 times more prevalent than* A. cryaerophilus* (140* versus* 36 strains, Table 4). Nevertheless, the true proportion of both species determined by direct culturing was 0.94 (102 of* A. butzleri versus* 109 strains of* A. cryaerophilus*). The former 3.9 proportion is in agreement with 4 times more prevalence of* A. butzleri* (248 strains) than* A. cryaerophilus* (60 strains) reported in a previous study in which samples were cultured using the same enrichment procedure but no direct plating [18]. A previous study on* Arcobacter* in broiler carcasses from Belgium compared the diversity of strains obtained by direct plating and by postenrichment in parallel and found that* A. butzleri* was 5.4 times (49 versus 9 strains) more prevalent than* A. cryaerophilus* by postenrichment culturing, while both species showed more similar proportion (42* A. butzleri* versus 31* A. cryaerophilus*, i.e., 1.4 : 1) when they were recovered by direct plating [16]. Consequently, those authors recommend the use of the two methods in parallel in order to enhance the diversity of species recovered. Another study from the same country performed by De Smet et al. [22] compared the recovered isolates from pig faeces using again the two methods. Regarding the species diversity, they found more isolates of* A. skirrowii* and* A. thereius* by direct plating than by postenrichment and more of* A. butzleri* and* A. trophiarum* by postenrichment than by direct plating [22]. Those authors hypothesized that the predominance of one species over another is due to the isolation procedure and medium used to recover the species rather than to its higher occurrence in the samples [17, 22]. More evidence of the influence of the culturing method applied has recently been provided by Fisher et al. [31], when studying the* Arcobacter* populations in wastewater from different cities in the United States and from the city of Reus (Spain) using a metagenomic analysis targeting the V4V5 regions of 16S rRNA gene. Those authors found that the predominant oligotypes matched with* A. cryaerophilus* while* A. butzleri* was only the eleventh most abundant oligotype [31]. Interestingly, they also report a correlation between the abundance of some* Arcobacter* oligotypes and water temperature. Another study on the* Arcobacter *diversity in shellfish and seawater observed that adding a 2.5% NaCl to the* Arcobacter*-CAT enrichment broth and subculturing in marine agar produced a significant increase on the recovered number of species (11 known species and 7 new candidate species) more than with the conventional method (7 known species and 2 new candidate species) [32]. Those authors recommended this new protocol for the isolation of* Arcobacter* from marine and brackish environments in order to avoid underestimation of the number of species [32]. They also stated that this simple modification of the culture shows a big influence on the community of species recovered. This finding is considered especially relevant in this metagenomics era, when it is not clear to what extent the differences observed between culturing and nonculturing methods are influenced by the culture media and conditions applied [32]. Therefore, future studies are warranted to assess the effect on* Arcobacter *isolation when using different media or conditions such as the incubation temperature. In this regard, the observed high prevalence and genetic diversity of* Arcobacter *spp. from wastewater confirm that this is an important reservoir for bacteria of this genus and could be a good matrix for testing different isolation protocols for the recovery of these bacteria.

## Figures and Tables

**Table 1:** Number of isolates and strains of the identified *Arcobacter* species found from wastewater using the two molecular methods (m-PCRand 16S rRNA-RFLP) in parallel.

Species	Number of isolates (%)	Number of strains (%)	% genetic diversity	Molecular identification
				m-PCR^a^/16S rRNA-RFLP^b^
*A. butzleri* (Ab)	354 (54.4)	247 (58.3)	69.8%	Ab/Ab
*A. cryaerophilus* (Ac)	226 (34.7)	150 (35.4)	66.4%	Ac/Ac
*A. thereius* (At)	37 (5.7)	9 (2.1)	24.3%	Ac/Ab
*A. skirrowii* (As)	16 (2.5)	5 (1.2)	31.3%	As/As
*A. *defluvii^c^ (Ad)	12 (1.8)	8 (1.9)	66.7%	~230 bp/Ad
*A. ellisii* (Ae)	3 (0.5)	2 (0.5)	66.7%	Ac/Ae
*A. *cloacae^d^ (Aclo)	2 (0.3)	2 (0.5)	100%	Ac/Aclo
*A. nitrofigilis* (An)	1 (0.2)	1 (0.2)	100%	As/An

Total	651	424	65.1%	

The isolates were genotyped with ERIC-PCR to determine the ones that showed the same ERIC-pattern and therefore belonged to the same strain. ^a^Houf et al. [19] and ^b^Figueras et al. [20]. Results of the RFLP were verified by sequencing the rpoB gene. ^c^New species recognized on the basis of the new RFLP pattern described by Figueras et al [20]. ^d^New species recognized on the basis of the new RFLP pattern and described by Levican et al. [21].

**Table 2:** *Arcobacter* species detected according to the method at the 5 sampling points in the wastewater treatment plant on the 6 different sampling occasions^a^.

Species detected		m-PCR		Culturing method	
		Direct	Postenrichment	Direct	Postenrichment
*A. butzleri* (Ab)		19 (100)	15 (100)	22 (78.6)	24 (82.8)
*A. cryaerophilus* (Ac)		10 (52.6)	9 (60)	27 (96.4)	16 (55.2)
*A. defluvii* (Ad)		0	0	2 (7.1)	3 (10.3)
*A. nitrofigilis* (Anit)		0	0	1 (3.6)	0
*A. cloacae* (Aclo)		0	0	0	2 (6.9)
*A. skirrowii* (As)		0	0	1 (3.6)	2 (6.9)
*A. thereius* (At)		0	0	3 (10.7)	4 (13.8)
*A. ellisii* (Ae)		0	0	2 (7.1)	0

Sampling month	Sampling point	m-PCR		Culturing method	
		Direct	Postenrichment	Direct	Postenrichment

April 2009	Influent water	Ac+Ab	ND	Ac+Anit	Ac+Ad+Aclo
	Primary sedimentation tank	Ac+Ab	ND	Ac+Ab+Ad	Ac+Ab
	Secondary bioreactor tank	Negative	ND	Ac+Ad	Ac+Ad
	Secondary sedimentation tank	Negative	ND	Negative	Ab+Ad
	Effluent water	Negative	ND	Negative	Negative

June 2009	Influent water	Negative	ND	Ac+Ab	Ab
	Primary sedimentation tank	Ab	ND	Ac+Ab	Ab
	Secondary bioreactor tank	Negative	ND	Ac+Ab	Ab
	Secondary sedimentation tank	Negative	ND	Ac	Ac+Ab
	Effluent water	Negative	ND	Ac+Ab	Ab

October 2009	Influent water	Ac+Ab	ND	Ac	Ac+Ab
	Primary sedimentation tank	Ac+Ab	ND	Ac+Ab	Ac+Ab
	Secondary bioreactor tank	Negative	ND	Ac	Ab
	Secondary sedimentation tank	Ab	ND	Ac+Ab	Ac+Ab
	Effluent water	Negative	ND	Ac+Ab	Ab

May 2011	Influent water	Ab	Ab+Ac	Ac+At	Ab+Ac+At
	Primary sedimentation tank	Ab+Ac	Ab+Ac	Ab+Ac+At	Ac+At
	Secondary bioreactor tank	Ab	Ab+Ac	Ab+Ac+As	Ac+As
	Secondary sedimentation tank	Ab	Ab+Ac	Ab+Ac	Ab+Ac
	Effluent water	Ab	Ab+Ac	Ab+Ac	Ab+Ac

June 2011	Influent water	Ab+Ac	Ab	Ab+Ac+Ae	Ab+As
	Primary sedimentation tank	Ab+Ac	Ab	Ab+Ac	Ab+Ac
	Secondary bioreactor tank	Ab	Ab	Ab+Ac+At	At
	Secondary sedimentation tank	Ab	Ab	Ab+Ac	Ab+Ac+At
	Effluent water	Ab	Ab	Ab+Ac	Ab

September 2011	Influent water	Ab+Ac	Ab+Ac	Ab+Ac+Ae	Ab+Aclo
	Primary sedimentation tank	Ab+Ac	Ab+Ac	Ab+Ac	Ab
	Secondary bioreactor tank	Ab+Ac	Ab+Ac	Ab+Ac	Ab+Ac
	Secondary sedimentation tank	Negative	Ab	Ab	Ab
	Effluent water	Negative	Ab+Ac	Ab+Ac	Ab+Ac

Total No. positive samples (%)		19/30 (63.3)	15/15 (100)	28/30 (86.7)	29/30 (86.7)

^a^The identified species are only mentioned once, independently of the number of strains obtained from each specific sample.

m-PCR: multiplex PCR Houf et al., 2000 [19]

ND: not done.

**Table 3:** Number of *Arcobacter* spp. strains recovered depending on aerobic (A) and microaerophilic (MA) incubation conditions.

Species^a^	Total recovered (%)	Only A (%)	Only MA (%)	A & MA (%)
*A. butzleri*	170 (60.7)	75 (58.6)	83 (65.4)	12 (48.0)
*A. cryaerophilus*	94 (33.6)	50 (39.1)	39 (30.7)	5 (20.0)
*A. thereius*	9 (3.2)	2 (1.6)	2 (1.6)	5 (20.0)
*A. skirrowii*	4 (1.4)	0	1 (0.8)	3 (12.0)
*A. ellisii*	2 (0.7)	1 (0.8)	1 (0.8)	0
*A. cloacae*	1 (0.4)	0	1 (0.8)	0

	280	128 (45.7%)	127 (45.4%)	25 (8.9%)

^a^The species *A. defluvii* and *A. nitrofigilis* do not appear at the table because they were isolated in 2009 where only aerobic incubation conditions were employed.

**Table 4:** Number of strains (%) of the different *Arcobacter* spp. recovered by direct plating (DP) and postenrichment (PE).

Species	Total recovered (%)	Only by DP (%)	Only by PE (%)	DP& PE (%)
*A. butzleri*	247 (58.3)	102 (46.8)	140 (74.1)	5 (29.4)
*A. cryaerophilus*	150 (35.4)	109 (50.0)	36 (19.0)	5 (29.4)
*A. thereius*	9 (2.1)	2 (0.9)	4 (2.1)	3 (17.6)
*A. skirrowii*	5 (1.2)	1 (0.5)	3 (1.6)	1 (5.9)
*A. defluvii*	8 (1.9)	1 (0.5)	4 (2.1)	3 (17.6)
*A. ellisii*	2 (0.5)	2 (0.9)	0	0
*A. cloacae*	2 (0.5)	0	2 (1.1)	0
*A. nitrofigilis*	1 (0.2)	1 (0.5)	0	0

	424	218 (51.4%)	189 (44.6%)	17 (4.0%)
